# Cooperative Goniopolar and Anomalous Nernst Transverse Thermoelectricity in Kagome Magnets

**DOI:** 10.1002/adma.74839

**Published:** 2026-08-27

**Authors:** Honghui Wang, Xiaolong Feng, Haihua Hu, Xuemin Shi, Ralf Koban, Walter Schnelle, Wenzhi Peng, Dazhi Hou, Jianjun Ying, Bin He, Yu Pan, Xiaoyuan Zhou, Claudia Felser

**Affiliations:** ^1^ Max Planck Institute for Chemical Physics of Solids Dresden Germany; ^2^ College of Physics and Center of Quantum Materials & Devices of Chongqing University Chongqing P. R. China; ^3^ CAS Key Laboratory of Strongly‐coupled Quantum Matter Physics, and Department of Physics University of Science and Technology of China Hefei Anhui P. R. China

**Keywords:** anomalous nernst, cooperative effect, goniopolar, kagome magnets

## Abstract

Transverse thermoelectrics (TEs), enabling interconversion between orthogonal heat and electric currents, offers unique advantages in device geometry and design flexibility. Yet, achieving large transverse TE signals at room temperature remains challenging. Here, we establish a cooperative framework in which the goniopolar effect and the anomalous Nernst effect jointly enhance the transverse TE response in kagome magnets. We demonstrate that kagome lattice‐derived electronic structures intrinsically yield axis‐dependent polar Seebeck coefficients arising from flat bands and van Hove singularities (VHSs), while broken time‐reversal symmetry induces topological band splitting that generates large net Berry curvature within the same energy window. Combining transport measurements, DFT calculations and real‐space thermal imaging, we demonstrate a room‐temperature record cooperative transverse TE signal of 15.6 µV/K in the kagome ferromagnet MgMn_6_Sn_6_ among metallic systems. Our findings establish a cooperative framework linking the anomalous Nernst and goniopolar effects and provide a widely applicable design strategy for achieving large transverse TE response in kagome magnets.

## Graphical abstract

A cooperative transverse thermoelectric mechanism is realized in kagome magnets, where axis‐dependent goniopolar effects and Berry curvature‐driven anomalous Nernst coexist within the same energy window. In MgMn_6_Sn_6_, this intrinsic synergy yields a record room‐temperature transverse thermopower of 15.6 µV K^−1^, establishing a general strategy for high‐performance transverse thermoelectrics without heterostructure engineering.

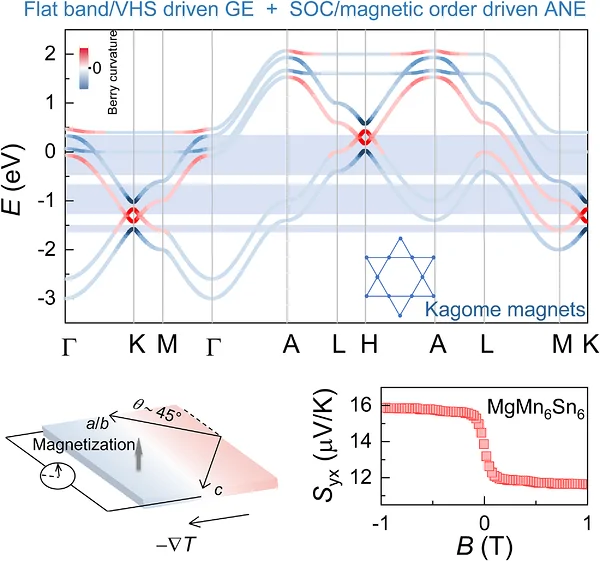

## 1 Introduction

Thermoelectric (TE) energy conversion has conventionally been implemented in longitudinal geometries, in which heat flow and electric current are aligned in the same direction. Although widely explored, such configurations impose constraints on device design, especially for harvesting heat from spatially non‐uniform or multidirectional sources. Transverse TEs, where heat flow and electric current are mutually orthogonal, offers an alternative route by geometrically decoupling heat flow from voltage output [1]. This configuration enables enhanced design flexibility, scalable coverage of complex thermal profiles, and reduced interfacial constrains. Achieving large transverse TE responses at room temperature is therefore highly desirable for applications ranging from waste‐heat harvesting and on‐chip cooling to spin‐caloritronics. With rapid advances in topological physics, transverse TE research has gained renewed attention. In magnetic topological materials, finite Berry curvature near the Fermi level generates an intrinsic anomalous Nernst response. Despite sustained efforts in band engineering and material discovery, room‐temperature anomalous Nernst coefficients in topological magnets typically remain below ∼ 6 µV/K [2, 3]. In parallel, goniopolar TE materials, characterized by axis‐dependent polar Seebeck coefficients, provide an alternative route to transverse TEs [4, 5, 6]. In such systems, rotational operations generate nonzero off‐diagonal components of the thermopower tensor, leading to a transverse response |*S*_yx_| = 1⁄2|*S*_yy_ − *S*_xx_|sin2*θ* [7], where *θ* is the rational angle (Figure 1a). However, experimentally realized goniopolar materials remain scarce. Whether these two mechanisms can coexist within a single material and cooperatively enhance transverse TEs, without relying on complicated heterostructures or multilayer engineering, remains an open question.

**FIGURE 1:**
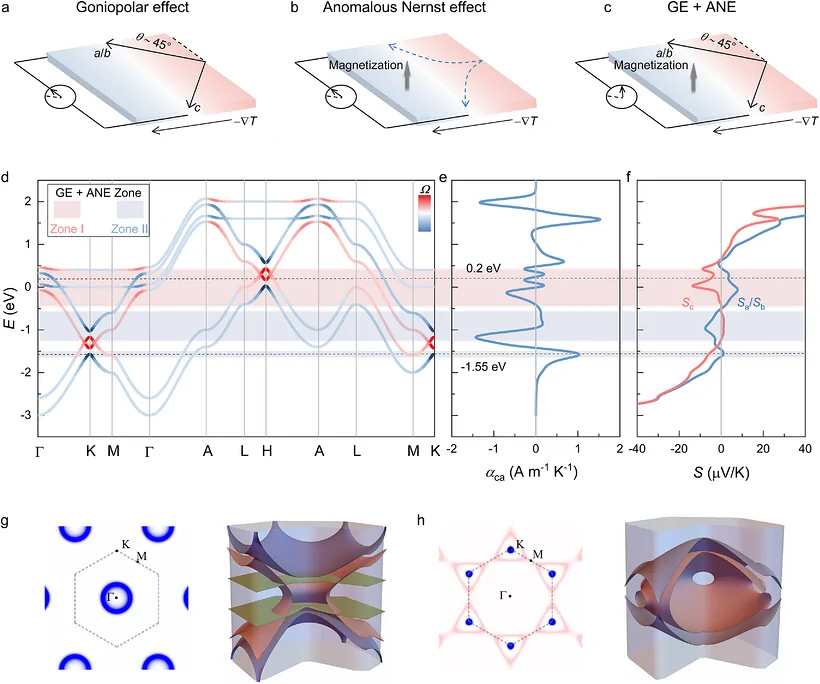
Measurement configurations of transverse TE effects and theoretical modelling of their coexistence. (a–c) Schematic measurement geometries for the goniopolar effect (GE, a), anomalous Nernst effect (ANE, b), and their cooperation (c). (d–h) Theoretical demonstration of the coexistence of goniopolar and anomalous Nernst effects in kagome magnets. (d) Electronic band structure with projected Berry curvature (*Ω*). (e) Corresponding energy‐resolved TE Hall conductivity. (f) Corresponding energy‐resolved axis‐dependent Seebeck coefficients. Model parameters: *λ*_km_ = 0.02 eV, *B* = 0.2 eV, *t*_i_ = −0.5 eV, and *t*_o_ = −0.4 eV. The shaded region highlights the energy window in which the anomalous Nernst and goniopolar effects coexist. (g and h) Representative energy at *E*_F_ = 0.2 eV (g) and −1.55 eV (h), showing the distribution of nontrivial Berry curvature in the *k*_z_ = 0 plane together with the corresponding three‐dimensional Fermi surface.

Herein, we demonstrate that kagome magnets provide a natural platform in which the goniopolar and anomalous Nernst effects emerge concurrently. We develop a general theoretical framework showing that flat bands and VHSs intrinsic to kagome lattices naturally give rise to axis‐dependent polar Seebeck coefficients, while the splitting of topological bands induced by broken time‐reversal symmetry contributes large net Berry curvature. Crucially, these two band features can coexist within the same energy window, enabling a cooperative enhancement of the transverse TE response. We experimentally validate this mechanism in the kagome ferromagnets RMn_6_Sn_6_ (R = Mg, Gd, and Tb). Among them, MgMn_6_Sn_6_ exhibits a record room‐temperature cooperative transverse thermopower of 15.6 µV K^−1^ among metallic systems, exceeding those of previously reported anomalous Nernst materials by a factor of 2.6 [2, 3]. This exceptional response originates from the combination of an intrinsic anomalous Nernst coefficient of ∼1.8 µV K^−1^ arising from Berry curvature and a large goniopolar transverse thermopower generated by a strongly anisotropic Fermi pocket derived from flat bands and VHSs together with four additional electron and hole pockets. These findings establish a cooperative design principle for realizing large transverse TE responses through the intrinsic integration of Berry curvature hotspots and axis‐dependent polar Seebeck coefficients within a single material platform.

## 2 Results and Discussions

### 2.1 Coexistence of Goniopolar and Anomalous Nernst Effects in Kagome Magnets

Kagome magnets constitute a particularly favorable platform for cooperative transverse TEs, owing to the coexistence of goniopolar and anomalous Nernst effects. From an electronic‐structure perspective, the intrinsic features of kagome bands naturally support this cooperation. Flat bands arising from destructive quantum interference, generate a high density of states and strongly anisotropic carrier transport [8]. In addition, VHSs enhance TE responses and give rise to mixed‐dimensional Fermi surfaces [9, 10]. These characteristics generate large anisotropic signals in longitudinal TE transport, providing an essential prerequisite for goniopolar TEs, with the potential polar Seebeck coefficients between in‐plane and out‐of‐plane crystallographic directions of the kagome lattice. Meanwhile, the coexistence of spin‐orbit coupling (SOC) and magnetic order reduces band degeneracies and gaps the Dirac points, generating nontrivial Berry‐curvature hotspots. These features are well‐known to produce sizable anomalous Hall and anomalous Nernst responses in kagome magnets [11, 12]. Therefore, kagome magnets offer a unique opportunity to simultaneously realize axis‐dependent polar Seebeck and intrinsic anomalous Nernst responses within a single material system.

To uncover the cooperative mechanism, we consider a single‐orbital kagome model given by

$$H=t\sum_{\langle \mathrm{ij}\rangle }c_{i}^{†}c_{j}+i\;\lambda_{\mathrm{km}}\sum_{\langle \langle \mathrm{ij}\rangle \rangle }v_{\mathrm{ij}}c_{i}^{†}\sigma_{z}c_{j}+\sum_{i}c_{i}^{†}(B\cdot \sigma )c_{i}$$

The first term describes nearest‐neighbor hopping between kagome sites. The second term introduces Kane‐Mele‐type SOC, which opens gap at the Dirac point and Γ points. The last term represents effective Zeeman splitting arising from magnetic exchange coupling, which breaks time‐reversal symmetry and enables intrinsic anomalous Nernst transport. A typical band structure with projected berry curvature is shown in Figure 1d, where SOC‐induced gaps are visible at the Γ and K points. The flat bands are slightly distorted due to the existence of SOC. For goniopolar transport, an anisotropic Fermi surface is the key requirement. Particularly in kagome materials, VHSs and flat bands has been identified as key fermiological features that can give rise to polar Seebeck response [13]. When the VHS is close to the Fermi level, the single hyperbolic Fermi surface produces pronounced polar Seebeck coefficients between in‐plane and out‐of‐plane directions (Figure 1f). Beyond the single‐band VHS scenario, goniopolar transport can also emerge from multiband contributions involving carriers of opposite polarity. A representative case arises when the flat band produces strongly directional transport along one direction, while dispersive bands hosting carriers of opposite polarity dominate conduction along other directions, together yielding axis‐dependent polar Seebeck coefficients. In contrast, anomalous Nernst transport requires large net berry curvature, which emerges once time‐reversal symmetry is broken. As showed in Figure 1d, large Berry curvature is strongly concentrated near SOC‐ and Zeeman effect‐split bands at the K and Γ points. A large intrinsic anomalous Nernst response is therefore expected when the Fermi surface samples these regions, consistent with the large TE Hall conductivity in Figure 1e. Notably, the Zeeman splitting has only a minor effect on Fermi surface geometry, preserving the conditions necessary for goniopolar transport.

Although the above model reproduces both anomalous Nernst and goniopolar transport individually, their cooperation arises naturally within specific energy windows determined by kagome electronic features. First, VHSs guarantee strongly anisotropic Fermi surfaces and therefore promote goniopolar transport. Although Berry curvature peaks near gaped Dirac points, the TE Hall conductivity is particularly sensitive to rapid changes in Berry curvature. Consequently, its maximum shifts away from the gapped Dirac points toward the VHS energy, aligning the anomalous Nernst response with the strong goniopolar effect, as shown for *E*_F_ = ‐1.55 eV in Figure 1d. Second, flat bands promote highly directional transport owing to their extreme anisotropy. When combined with finite berry curvature, they further reinforce the TE Hall conductivity. Thus, the anomalous Nernst and goniopolar responses are simultaneously guaranteed upon introducing additional bands hosting carriers of opposite polarity, as illustrated for *E*_F_ = 0.2 eV in Figure 1d. Because these features can coexist within the same energy window in multi‐orbital kagome magnets, they facilitate the proposed cooperative enhancement between large intrinsic anomalous Nernst and goniopolar effects.

### 2.2 Cooperative Goniopolar and Anomalous Nernst Transverse TEs in Kagome Ferromagnet MgMn_6_Sn_6_

Mn‐based kagome magnets typically exhibit relatively high Curie temperatures due to Mn 3*d* electrons [14], making them ideal platforms for room‐temperature transverse TEs. We first selected the kagome ferromagnet MgMn_6_Sn_6_ as an experimental platform to examine cooperative transverse TE transport. MgMn_6_Sn_6_ undergoes a ferromagnetic transition at ∼ 321 K (Figure S2), with the easy magnetization axis lying within the *ab* plane. Density functional theory calculations (Figure 2a) reveal that electronic states near the Fermi level are dominated by Mn 3*d* orbitals, forming a multi‐orbital electronic structure derived from kagome lattice. The calculated band structure exhibits several characteristic features near *E*_F_, including flat bands derived from Mn *d* orbitals, VHSs at the M point and additional nodal points. Incorporating SOC in the presence of ferromagnetic order reduces band degeneracies and generates large net Berry curvature, which produces strong TE Hall conductivity (Figure 2c) and a pronounced anomalous Nernst response below the magnetic ordering temperature. The anomalous Nernst coefficient peaks at 2.6 µV/K near 240 K (Figure S3b) and reaches 1.8 µV/K at 300 K (Figure 2d), comparable to the highest values reported in other kagome magnets (Figure S3d) [11, 15, 16, 17]. According to linear response theory, the Nernst coefficient is given by *S*_yx_ = *α*_yx_ *ρ*_yy_ − *σ*_yx_ *ρ*_yy_ *S*_xx_ [3], where *α*_yx_, *ρ*_yy_ and *σ*_yx_ are the TE Hall conductivity, longitudinal resistivity and Hall conductivity, respectively. The first term dominates in MgMn_6_Sn_6_ (Figure S4), indicating that the anomalous transverse TE response primarily originates from intrinsic Berry curvature contributions. The experimentally extracted TE Hall conductivity reaches 1.25 A m^−1^ K^−1^, in agreement with theoretical calculations (Figure S5). Comparisons with other high‐Curie temperature kagome magnets and conventional ferromagnets (Figure S3d) further confirm the intrinsic anomalous Nernst response in MgMn_6_Sn_6_. These results establish a robust Berry‐curvature‐driven transverse TE channel, providing the first essential component for cooperative transverse TEs.

**FIGURE 2:**
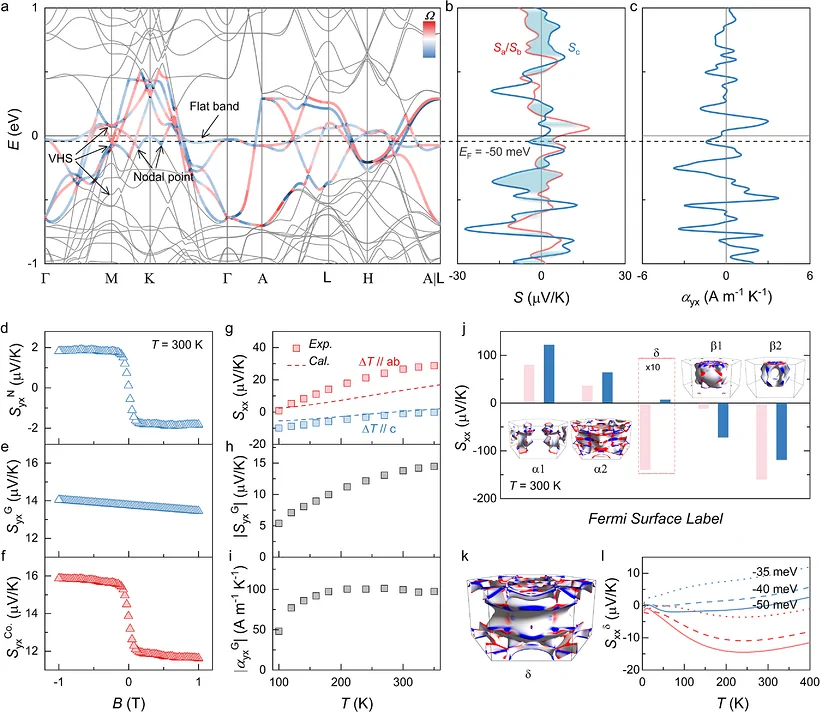
Cooperative transverse TEs in kagome ferromagnet MgMn_6_Sn_6_. (a) Calculated electronic band structure of MgMn_6_Sn_6_. (b) Corresponding energy‐resolved axis‐dependent Seebeck coefficients. The shaded region highlights the energy window where the goniopolar effect generally exists. (c) Corresponding energy‐resolved TE Hall conductivity derived from Berry curvature. (d–f) Field‐dependent room‐temperature anomalous Nernst (d), goniopolar (e) and their cooperative signal (f), respectively. (g) Temperature‐dependent axial Seebeck coefficients with the heat flow along in‐plane and out‐of‐plane directions, respectively. Experimental measurement (solid dots) and DFT calculations (dash lines) with *E*_F_ = ‐50 meV exhibit excellent consistent. (h) Temperature‐dependent goniopolar thermopower. (i) Temperature‐dependent goniopolar TE Hall conductivity. (j) Fermi pockets‐resolved axial Seebeck coefficients calculated at 300 K. (k) Fermi pocket δ associated with flat bands and van Hove singularities. It can alone yield the goniopolar effect. (l) Calculated temperature‐dependent axial Seebeck coefficient for Fermi pocket δ at *E*_F_ = −35, −40 and −50 meV.

In addition to Berry‐curvature‐driven anomalous Nernst transport, MgMn_6_Sn_6_ also hosts electronic features favourable for goniopolar TEs. The band structure reveals a nearly flat band located ∼ −50 meV along the Γ‐K direction, mainly derived from Mn *d*_xz_ and *d*_yz_ orbitals. This flat band is accompanied by VHSs at the M point, where saddle‐point dispersions generate pronounced peaks in the density of states. Notably, two types of VHSs with opposite concavity coexist, giving rise to strong energy‐dependent anisotropic longitudinal TE transport (Figure 2b). The energetic proximity of these kagome lattice‐derived electronic features to the Fermi level naturally produces axis‐dependent polar Seebeck coefficients over a broad energy window, establishing the second prerequisite for cooperative transverse TEs. To directly probe the goniopolar response, temperature‐dependent Seebeck coefficients are measured along different crystallographic directions (Figure 2g). Significant axis‐dependent polar Seebeck coefficients are observed, with positive thermopower within the *ab* plane and negative thermopower along the *c* axis over a wide temperature range from 100 to 350 K. To maximize the goniopolar response, the heat flow is rotated by an angle *Θ* from the *ab* plane toward the *c* axis. The optimal rotation angle is given by cos2*Θ* = [(1 + (*κ*_cc_/*κ*_aa_/*ρ*_cc_/*ρ*_aa_)1/2]^−1^ [7], where *κ* and *ρ* is thermal conductivity and resistivity, respectively. Owing to the dominant electronic contribution to thermal conductivity (Figure S6), the optimal angle is close to 45°. Under this configuration, the absolute goniopolar thermopower reaches 5 to 15 µV/K between 100 and 350 K (Figure 2h) and generates a TE Hall conductivity of ∼ 100 A m^−1^ K^−1^ (Figure 2i), representing a room‐temperature record among known materials. Goniopolar TEs is generally due to single‐band and multiband mechanisms [18]. To clarify the microscopic origin of the large goniopolar response in MgMn_6_Sn_6_, we conducted a thorough analysis of the Fermi surface. The calculated response agrees with the experimental observations when the Fermi level is shifted to −50 meV (Figure 2g). Five bands crossing the Fermi level contribute to transport (Figures 2j,k, Figure S7): two electron pockets (α1 and α2), two‐hole pockets (β1 and β2), and one strongly anisotropic pocket (δ). The combined multiband contributions produce a large goniopolar response. Notably, the highly anisotropic δ pocket alone can support goniopolar transport (Figure 2l), arising from flat bands and VHSs associated with Mn *d*_xz_ and *d*_yz_ orbitals (Figure S9).

Crucially, the simultaneous presence of strong Berry curvature‐driven anomalous Nernst transport and pronounced goniopolar effect enables their cooperation. As shown in Figure 2d–f, the combined contributions produce a record room‐temperature transverse TE signal of 15.6 µV/K, exceeding previously reported values in topological magnetic systems [11, 15, 16, 17, 19, 20]. The cooperative effect is further observed in TbMn_6_Sn_6_ and GdMn_6_Sn_6_, which exhibit transverse thermoelectric signals of 13.8 µV K^−1^ and 9.7 µV K^−1^ (Figures S10–S13), respectively. These results demonstrate that kagome electronic structures intrinsically integrate the two essential mechanisms and provide direct experimental validation of cooperative transverse TEs.

### 2.3 Thermal Imaging and Transverse TE Cooling in MgMn_6_Sn_6_

To directly visualize the cooperative mechanism and validate the device configuration proposed in Figure 1c, we performed real‐space thermal imaging combined with measurements of transverse TE cooling, including the Ettingshausen and transverse Peltier effects. Lock‐in thermal imaging at 300 K was conducted under an alternating electric current at zero magnetic field and the detailed experimental setup is similar to those in our previous work [21]. When the current was applied along the longitudinal direction, a pronounced temperature modulation developed along the transverse direction, demonstrating efficient conversion between longitudinal electric current and transverse heat flow. Figure 3b,c show the lock‐in amplitude (*A*) and phase (*φ*) images, respectively. A clear transverse temperature modulation emerges upon current injections, with opposite sample edges exhibiting contrasting thermal responses. The spatial distribution of the temperature‐difference signal is more clearly visualized in Figure 3d, which presents the in‐phase temperature component *X* = *A*cos*φ*. For quantitative analysis, two regions of interest (regions I and II) near the transverse edges of the sample were selected. The electric current (*J*) dependence of the amplitude *X* for these regions is shown in Figure 3e, where the plotted values represent spatial averages within each area. The amplitude scales linearly with *J*, while the phase remains independent of *J* and exhibits a 180° phase difference between regions I and II. This linear behavior confirms that the temperature modulation originates from the transverse Peltier effect rather than Joule heating (Figure 3e; Figures S8 and S15). To further probe cooperative transverse TE transport, magnetic‐field‐dependent transport measurements were also performed. Upon applying a magnetic field of approximately ‐0.2 T (inset of Figure 3f), the transverse temperature difference increases sharply. The enhancement follows the same scaling behavior as the cooperative transverse TE signal shown in Figure 2f, indicating that magnetic‐field‐induced anomalous Ettingshausen contributions constructively reinforce the transverse response. Consequently, cooperation between the inverse Nernst effect (Ettingshausen effect) and the inverse goniopolar effect (transverse Peltier effect) yields amplified transverse cooling functionality. These results validate the cooperative device concept and provide direct experimental evidence that Ettingshausen and transverse Peltier effects can synergistically enhance transverse TE cooling capacity in kagome magnets.

**FIGURE 3:**
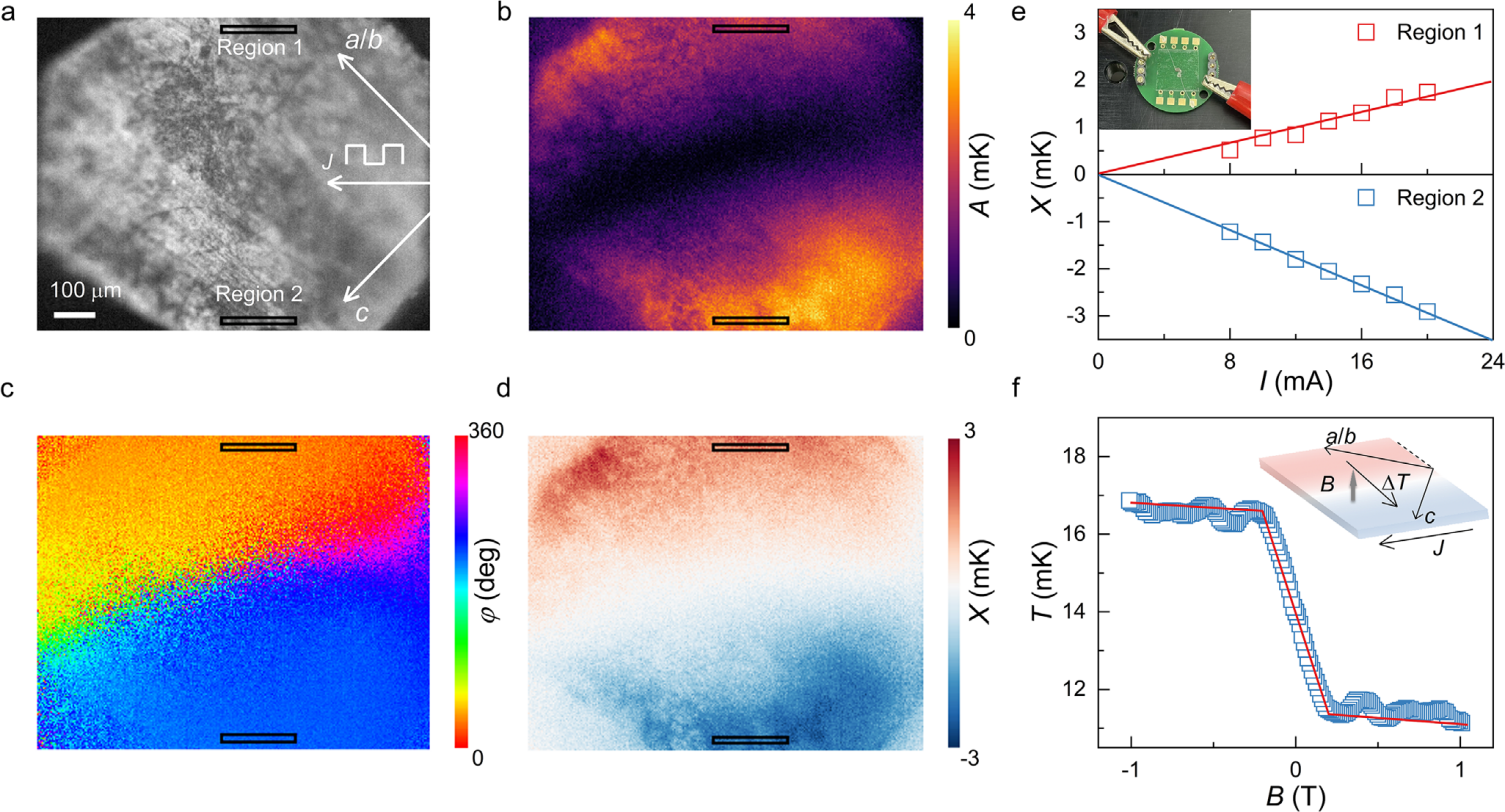
Thermal imaging and transverse TE cooling. (a) Optical image of the sample used for thermal imaging measurements. (b and c) Lock‐in amplitude (*A*) and phase (*φ*) images acquired under an alternating longitudinal current *J* (amplitude, 20 mA; frequency, 2.5 Hz) applied over 2000 cycles. (d) Spatial map of the in‐phase thermal response, defined as *X* = *A*cos*φ*. (e) Electric current (*J*) dependence of the transverse thermal response *X*, extracted from two regions of interest (region 1 and region 2) marked by the rectangles in (d). (f) Magnetic‐field dependence of the cooling temperature difference measured at 300 K. The transport measurement configuration is shown in the inset.

### 2.4 Outstanding Transverse Thermoelectric Performance Enabled by Cooperative Effect

Having clarified the microscopic origin and experimentally validated cooperative transverse TEs in MgMn_6_Sn_6_, we now evaluate its performance and broader implications. The simultaneous presence of anomalous Nernst and goniopolar effects produces a transverse TE response that exceeds what either mechanism can achieve alone. This enhancement originates from the constructive addition of two distinct contributions that are naturally aligned in both energy and geometry. The anomalous Nernst component arises from Berry‐curvature hotspots generated by spin‐orbit coupling and magnetic order. In parallel, flat bands and VHSs near the Fermi level give rise to strong axis‐dependent polar Seebeck coefficients, enabling a sizable goniopolar thermopower. Because these two mechanisms coexist within overlapping energy windows and share the same polarity under a unified device configuration, their transverse contributions reinforce the overall response. This cooperative alignment directly accounts for the large transverse TE output observed in MgMn_6_Sn_6_.

To quantify the performance gain, we benchmark MgMn_6_Sn_6_, TbMn_6_Sn_6,_ and GdMn_6_Sn_6_ against representative materials exhibiting strong anomalous Nernst or goniopolar responses [2, 3, 6, 7, 11, 15, 16, 19, 20, 22, 23, 24, 25, 26, 27, 28]. As shown in Figure 4a,b, both the room‐temperature transverse thermopower and TE Hall conductivity surpass those of previously reported transverse TE systems. The transverse thermopower reaches 15.6 µV/K at 300 K, significantly exceeding those of typical anomalous Nernst and metallic goniopolar materials. At the same time, the TE Hall conductivity is markedly enhanced relative to known transverse TE compounds. The simultaneous improvement of these two key parameters underscores the advantage of integrating Berry curvature‐driven and goniopolarity‐driven mechanisms within a single material. We further evaluate the TE figure of merit (*ZT*). As shown in Figure 4c, the cooperative goniopolar‐anomalous Nernst mechanism yields transverse *ZT* values that significantly surpass those of existing transverse TE materials operating near room temperature. These results demonstrate that cooperative transverse TEs provides an effective route to overcome the long‐standing limitation of weak transverse TE efficiency at room temperature and establishes a general design strategy for high‐performance transverse TE materials. Notably, this enhanced performance is achieved without interface engineering, multilayer stacking, or device‐level optimization. The enhancement derives purely from intrinsic bulk electronic structure characteristics of the kagome lattice. Unlike conventional approaches that aim to independently maximize Berry curvature or Seebeck anisotropy, the present strategy exploits their natural coexistence, thus establishing a general design principle for achieving high‐performance transverse TE materials.

**FIGURE 4:**
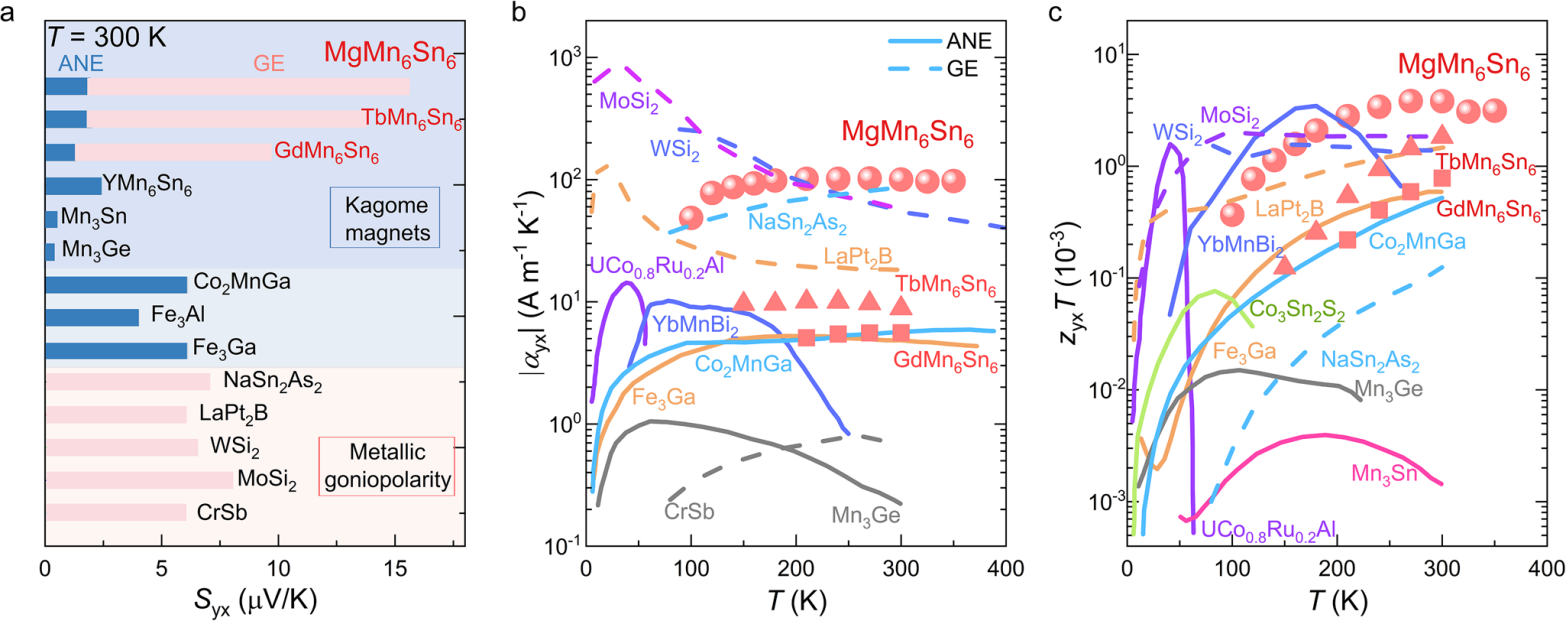
Outstanding transverse TE performance enabled by cooperative effect. (a–c) Comparison of the transverse thermopower (a), TE Hall conductivity (b), and transverse TE figure of merit (c) of MgMn_6_Sn_6_ with those of representative materials exhibiting strong Nernst effect and reported metallic goniopolar materials.

## 3 Conclusion

In summary, we demonstrate that transverse TEs can be significantly enhanced through a cooperative mechanism that integrates Berry curvature‐driven anomalous Nernst transport with axis‐dependent polar Seebeck responses. Taking the kagome ferromagnet RMn_6_Sn_6_ (R = Mg, Gd, and Tb) as a model system, we experimentally verify that these two mechanisms can coexist within a single material and constructively reinforce the transverse TE signal. Beyond establishing MgMn_6_Sn_6_ as a high‐performance platform, our results reveal a general design principle for transverse TEs: large responses can emerge when Berry curvature hotspots and strongly anisotropic thermopower coexist within the same energy window. Such energetic alignment enables their transverse contributions to add constructively under a unified device geometry. Looking forward, this cooperative strategy provides multiple routes for further optimization. Because both the anomalous Nernst and goniopolar responses are highly sensitive to the Fermi level, tuning carrier concentration through chemical substitution, electrostatic gating, or pressure may further amplify the cooperative signal. From an application perspective, integrating transverse thermoelectric materials with permanent magnets, or alternatively developing hard magnetic materials with high Curie temperatures, will be crucial for realizing robust and energy‐efficient device operation without external magnetic fields. In addition, exploring new material platforms, such as kagome magnets with stronger spin‐orbit coupling or other topological magnetic systems with highly anisotropic electronic structures may enable even larger transverse TE performance. Overall, cooperative transverse TEs offers a promising paradigm for next‐generation TE materials and opens new opportunities for efficient room‐temperature energy conversion and solid‐state cooling technologies.

## 4 Experimental Section/Methods

### 4.1 Crystal Growth and Transport Measurement

Single crystals were synthesized via the flux method [29]. After the growth, the quality and orientation of the single crystals were characterized via x‐ray diffraction (Figure S1) and structural refinement (Tables S1 and S2). Nernst thermopowers, Ettingshausen temperature differences, Seebeck coefficients, and thermal conductivity were measured under high vacuum using a steady‐state technique (Figure S16) on a Physical Property Measurement System (PPMS‐9T, Quantum Design).

### 4.2 Theoretical Models and Calculations

Density functional theory (DFT) calculations were conducted using the Vienna ab initio simulation package (VASP) based on the projector augmented wave method (PAW) [30, 31, 32], where the generalized gradient approximation (GGA) of the Perdew–Burke–Ernzerhof (PBE)‐type exchange‐correlation potential is adopted [33]. A 9 × 9 × 4 Γ‐centered k‐mesh of Monkhorst–Pack sampling is applied with a plane‐wave energy cutoff of 480 eV. The energy and force convergence criteria were set to 10^−8^ eV and 10^−3^ eV/Å, respectively. Maximally localized Wannier functions (MLWFs) are obtained through the Wannier90 code [34], by including the Mn *s*, *d*, and Sn *p* orbitals. WannierTools [35] is used to calculate the berry curvature, and thermoelectric Hall conductivity. Notably, in addition to MgMn_6_Sn_6_, TbMn_6_Sn_6_ and GdMn_6_Sn_6_, the electronic band structures, axis‐dependent thermopower, and thermoelectric Hall conductivity of LiMn_6_Sn_6_ are also shown in Figure S14.

### 4.3 Thermal Imaging

Imaging of the transverse TE cooling was measured by lock‐in thermography (LIT) [21]. The LIT system is composed of a mid‐infrared camera, a microscope lens, a system processing unit that performs real‐time Fourier analysis of detected thermal images and a source meter.

## Author Contributions

Claudia Felser and Xiaoyuan Zhou supervised the project. Honghui Wang conducted the transport measurement. Xiaolong Feng performed theoretical calculations. Jianjun Ying, Dazhi Hou, and Wenzhi Peng measured the thermal imaging. Bin He provided valuable input during the discussion of results. Honghui Wang analyzed the data. Honghui Wang wrote the manuscript with contributions and critical feedback from all authors.

## Funding

The National Key R&D Program of China (2023yfb3809500), the National Natural Science Foundation of China (Grant Nos. 52125103 and 12595331), the Deutsche Forschungsgemeinschaft (DFG, German Research Foundation) through the Würzburg‐Dresden Cluster of Excellence ctd.qmat‐Complexity, Topology and Dynamics in Quantum Matter (EXC 2147, grant 390858490).

## Conflicts of Interest

The authors declare no conflicts of interest.

## Supporting information




**Supporting File**: adma74839‐sup‐0001‐SuppMat.docx.

## Data Availability

The data that support the findings of this study are available from the corresponding author upon reasonable request.
