# Supplementary material for: Cooperative Goniopolar and Anomalous Nernst Transverse Thermoelectricity in Kagome Magnets
Source: Adv Mater. 2026 Aug 27;38(59):e74839. doi: 10.1002/adma.74839 (PMC13689901; doi:10.1002/adma.74839)
Supplement: Supplementary file 1 [file ADMA-38-e74839-s001.docx]

Supporting Information

Title: **Cooperative goniopolar and anomalous Nernst transverse thermoelectricity in kagome magnets**

Honghui Wang,* Xiaolong Feng,* Haihua Hu, Xuemin Shi, Ralf Koban, Walter Schnelle, Wenzhi Peng, Dazhi Hou, Jianjun Ying,* Bin He, Yu Pan, Xiaoyuan Zhou,* Claudia Felser*

**CONTENTS**

**Supplementary 1**: XRD in MgMn_6_Sn_6_

**Supplementary 2**: Magnetization in MgMn_6_Sn_6_

**Supplementary 3**: Anomalous Hall and Nernst effects in MgMn_6_Sn_6_

**Supplementary 4**: The *α*_yx_*ρ*_yy_ term dominates anomalous Nernst effect in MgMn_6_Sn_6_

**Supplementary 5**: Berry curvature induced large anomalous Nernst signal in MgMn_6_Sn_6_

**Supplementary 6**: Anisotropic resistivity and thermal conductivity in MgMn_6_Sn_6_

**Supplementary 7**: Calculated temperature-dependent axial Seebeck coefficients in MgMn_6_Sn_6_

**Supplementary 8**: Thermal imaging of Joule heating in MgMn_6_Sn_6_

**Supplementary 9**: Orbital-resolved electronic band structure and density of states in MgMn_6_Sn_6_

**Supplementary 10**: Cooperative transverse thermoelectricity in TbMn_6_Sn_6_

**Supplementary 11**: Cooperative transverse thermoelectricity in GdMn_6_Sn_6_

**Supplementary 12**: Cooperative transverse thermoelectricity in LiMn_6_Sn_6_

**Supplementary 13**: Comparison between the Peltier Effect and Joule Heating

**Supplementary 14**: Experimental configuration for thermoelectric transport measurements

**References**

**Supplementary 1: XRD and structural refinement in MgMn_6_Sn_6_**

Structural refinement of MgMn_6_Sn_6_, and the detailed refinement parameters are summarized in **Table R1-2**. When the site occupancies were allowed to vary during the refinement, they were found to be in the range of 0.99~1.02, which is in excellent agreement with the EDS analysis, yielding an elemental ratio of Mg:Mn:Sn = 1:5.95:5.98.

**Fig. S1** Single-crystal X-ray diffraction (XRD) pattern of MgMn_6_Sn_6_. The presence of only sharp (00l) diffraction peaks confirms the high quality of MgMn_6_Sn_6_. The inset shows the sample used for transport measurements.

| **Table R1 Crystal data and structure refinement for MgMn_6_Sn_6_.** | |
| --- | --- |
| Empirical formula | MgMn_6_Sn_6_ |
| Formula weight | 1066.09 |
| Temperature/K | 299.89(10) |
| Crystal system | hexagonal |
| Space group | P6/mmm |
| a/Å | 5.52100(10) |
| b/Å | 5.52100(10) |
| c/Å | 9.0413(2) |
| α/° | 90 |
| β/° | 90 |
| γ/° | 120 |
| Volume/Å^3^ | 238.669(10) |
| Z | 1 |
| ρ_calc_g/cm^3^ | 7.417 |
| μ/mm^‑1^ | 184.695 |
| F(000) | 462.0 |
| Crystal size/mm^3^ | 0.073 × 0.048 × 0.017 |
| Radiation | Cu Kα (λ = 1.54184) |
| 2Θ range for data collection/° | 9.782 to 158.426 |
| Index ranges | -6 ≤ h ≤ 6, -7 ≤ k ≤ 6, -11 ≤ l ≤ 10 |
| Reflections collected | 1218 |
| Independent reflections | 141 [R_int_ = 0.0928, R_sigma_ = 0.0329] |
| Data/restraints/parameters | 141/0/14 |
| Goodness-of-fit on F^2^ | 1.273 |
| Final R indexes [I>=2σ (I)] | R_1_ = 0.0397, wR_2_ = 0.0985 |
| Final R indexes [all data] | R_1_ = 0.0397, wR_2_ = 0.0985 |
| Largest diff. peak/hole / e Å^-3^ | 1.66/-2.39 |

| **Table R2: Fractional Atomic Coordinates and Equivalent Isotropic Displacement Parameters. U_eq_ is defined as 1/3 of the trace of the orthogonalised U_IJ_ tensor.** | | | | | | |
| --- | --- | --- | --- | --- | --- | --- |
| Atom | Wyckoff site | x | y | z | Occ. | U_eq_ |
| Mg | 1a | 0.666667 | 0.333333 | 1.000000 | 1 | 0.0159111 |
| Mn | 6i | 0.666667 | 0.333333 | 0.500000 | 1 | 0.0164667 |
| Sn1 | 2c | 1.000000 | 1.000000 | 0.827710 | 1 | 0.0175444 |
| Sn2 | 2d | 0.500000 | 0.500000 | 0.748650 | 1 | 0.0150111 |
| Sn3 | 2e | 1.000000 | 1.000000 | 0.500000 | 1 | 0.0393333 |

**Supplementary 2: Magnetization in MgMn_6_Sn_6_**

**Fig. S2 a**, Temperature dependence of the field-cooled (FC) and zero-field-cooled (ZFC) magnetization *M* for *B* || *ab* plane (blue) under an applied field of *B* = 0.01 T. **b**, Field dependence of the magnetization *M* at *T* = 5 K for *B* || *ab* (red) and *B* || *c* (blue), respectively, indicating that the magnetic easy axis lies in the *ab* plane.

**Supplementary 3: Anomalous Hall and Nernst effects in MgMn_6_Sn_6_**

**Fig. S3 a**-**b**, Magnetic field-dependent anomalous Hall resistivity and anomalous Nernst thermopower in the temperature range from 100 K to 300 K. **c**, The distribution of Berry curvature. **d**, Comparison of the anomalous Nernst thermopower with those of representative magnetic kagome materials and conventional ferromagnets.^1-4^

**Supplementary 4: The *α*_yx_*ρ*_yy_ term dominates anomalous Nernst effect in MgMn_6_Sn_6_**

**Fig. S4** Temperature dependence of *α*_yx_*ρ*_yy_ (**a**) and *σ*_yx_*ρ*_yy_*S*_xx_ (**b**). The anomalous Nernst thermopower is given by the sum of the two terms, *S*_yx_ = *α*_yx_*ρ*_yy_ − *σ*_yx_*ρ*_yy_*S*_xx_. The *α*_yx_*ρ*_yy_ term dominates the anomalous Nernst signal.

**Supplementary 5: Berry curvature induced large anomalous Nernst signal in MgMn_6_Sn_6_**

**Fig. S5 a**, Temperature dependence of thermoelectric Hall conductivity obtained by theoretical calculation and formula derivation (*α*_yx_ = *S*_yx_/*ρ*_yy_ + *σ*_yx_*S*_xx_). The deviation between the calculated intrinsic thermoelectric Hall conductivity and the experimentally determined value may arise from extrinsic scattering contributions, including side-jump and skew-scattering mechanisms. **b**, Temperature dependence of anomalous Nernst thermopowr at *B* = 0.2 T. Notably, *α*_yx_^N^(*T*) and *S*_yx_^N^(*T*) exhibit similar behavior, further confirming that the anomalous Nernst signal is due to intrinsic band contribution.

**Supplementary 6: Anisotropic resistivity and thermal conductivity in MgMn_6_Sn_6_**

**Fig. S6 a-b**, Temperature-dependent resistivity (**a**) and thermal conductivity (**b**) with electric and heat currents applied along *ab* plane and *c* axis, respectively. The residual resistivity ratio, *RRR* = *ρ*_ab_(300 K)/*ρ*_ab_(2 K) ≈ 203, indicates the high quality of the single crystal. The electronic thermal conductivity is obtained through Wiedemann-Franz law *κ*_e_ = *L*_0_*T*/*ρ* and the phonon thermal conductivity is obtained through formula *κ*_ph_ = *κ*_total_ - *κ*_e_.

**Supplementary 7: Calculated temperature-dependent axial Seebeck coefficients in MgMn_6_Sn_6_**

**Fig. S7 a**, **c**, **e**, **g**, Nearly isotropic Fermi pockets at *E*_F_ = -50 meV. **b**, **d**, **f**, **h,** Calculated temperature dependence of Seebeck coefficients.

**Supplementary 8: Thermal imaging of Joule heating in MgMn_6_Sn_6_**

**Fig. S8 a**, Optical image of the sample used for thermal imaging measurements. **b**-**c**, Lock-in amplitude (*A*) and phase (*φ*) images acquired under an alternating longitudinal current *J* (*J*_max_ = 20 mA; *J*_min_ = 0 mA; frequency, 2.5 Hz) applied over 2000 cycles. **d**, Spatial map of the in-phase thermal response, defined as *X* = *A*cos*φ*. **e**-**f**, *J^2^* dependence of the transverse thermal response *A* and phase *φ*, extracted from two regions of interest (region 1 and region 2) marked by the rectangles in **d**. Notably, the longitudinal Joule heat flow due to contact resistance generates negligible temperature differences in the transverse direction, further indicating that the transverse thermoelectric signal originates from the goniopolar effect.

**Supplementary 9: Orbital-resolved electronic band structure and density of states in MgMn_6_Sn_6_**

**Fig. S9** Orbital-resolved electronic band structure and density of states (*DOS*).

**Supplementary 10: Cooperative transverse thermoelectricity in TbMn_6_Sn_6_**

**Fig. S10** **a**, Calculated electronic band structure of TbMn_6_Sn_6_. **b**, Corresponding energy-resolved axis-dependent Seebeck coefficients. The shaded region highlights the energy window where the goniopolar effect generally exists. **c**, Corresponding energy-resolved TE Hall conductivity derived from Berry curvature.

**Fig. S11** **a**, **c** and **d** Temperature-dependent axial Seebeck coefficient, resistivity and thermal conductivity. The dash line in **a** represents the calculated Seebeck coefficient with the Fermi level shifted to *E*_F_ = −40 meV. **b**, Field-dependent the cooperative transverse thermopower.

**Supplementary 11: Cooperative transverse thermoelectricity in GdMn_6_Sn_6_**

**Fig. S12 a**, Calculated electronic band structure of GdMn_6_Sn_6_. **b**, Corresponding energy-resolved axis-dependent Seebeck coefficients. The shaded region highlights the energy window where the goniopolar effect generally exists. **c**, Corresponding energy-resolved TE Hall conductivity derived from Berry curvature.

**Fig. S13** **a**, **c** and **d** Temperature-dependent axial Seebeck coefficient, resistivity and thermal conductivity. The dash line in **a** represents the calculated Seebeck coefficient with the Fermi level shifted to *E*_F_ = −50 meV. **b** Field-dependent the cooperative transverse thermopower.

**Supplementary 12: Cooperative transverse thermoelectricity in LiMn_6_Sn_6_**

**Fig. S14** **a**, Calculated electronic band structure of LiMn_6_Sn_6_. **b**, Corresponding energy-resolved axis-dependent Seebeck coefficients. The shaded region highlights the energy window where the goniopolar effect generally exists. **c**, Corresponding energy-resolved TE Hall conductivity derived from Berry curvature.

**Supplementary 13**: Comparison between the Peltier Effect and Joule Heating

**Fig.S15** Comparison between Peltier heat and Joule heating. **a**, Current-dependent Peltier heat and Joule heating at 300 K. The sample dimensions are approximately 1 mm × 1 mm × 1 mm. **b**, Temperature-dependent Peltier heat and Joule heating under a fixed current of 20 mA.

**Supplementary 14**: Experimental configuration for thermoelectric transport measurements

Fig.S16 **Experimental configurations for thermoelectric and inverse thermoelectric measurements.** **a**, Schematic of the setup used for thermal transport and thermoelectric characterization. One end of the sample was thermally anchored to a copper heat sink, while the opposite end was connected to a copper thermal reservoir equipped with a resistive heater to generate a controlled temperature gradient. The longitudinal temperature difference was monitored using a differential thermocouple attached along the sample length. Electrical contacts were arranged to simultaneously measure the Seebeck, Nernst, and goniopolar voltages. **b**, Configuration employed for inverse thermoelectric measurements. The sample was mounted in a geometry similar to that used for thermoelectric characterization, with the temperature difference monitored by the same differential thermocouple arrangement. In this case, an electrical current was driven along the transverse direction, and the resulting temperature gradient was detected along the longitudinal direction. To minimize current inhomogeneity, silver paste was uniformly coated on the opposing transverse surfaces, ensuring a nearly uniform current injection across the sample cross-section.

**References**

1. M. Ikhlas, et al. Large anomalous Nernst effect at room temperature in a chiral antiferromagnet. *Nat. Phys.* **13**, 1085-1090 (2017).
2. Wuttke, C. et al. Berry curvature unravelled by the anomalous Nernst effect in Mn_3_Ge. *Phys. Rev. B* **100**, 085111 (2019).
3. Zhang, H., Xu, C. Q., Ke, X. Topological Nernst effect, anomalous Nernst effect, and anomalous thermal Hall effect in the Dirac semimetal Fe_3_Sn_2_. *Phys. Rev. B* **103**, L201101 (2021).
4. Xu, X. et al. Topological charge-entropy scaling in kagome Chern magnet TbMn_6_Sn_6_. *Nat. Commun.* **13**, 1197 (2022).
